# Role of Dithiothreitol in Detection of Orthopaedic Implant-Associated Infections

**DOI:** 10.3390/jpm14040334

**Published:** 2024-03-22

**Authors:** Matthaios Bakalakos, Christos Vlachos, Margarita-Michaela Ampadiotaki, Antonios Stylianakis, Nikolaos Sipsas, Spiros Pneumaticos, John Vlamis

**Affiliations:** 1 3rd Orthopaedic Department, National and Kapodistrian University of Athens, KAT General Hospital, 14561 Athens, Greece; christosorto@gmail.com (C.V.); spirosgp@med.uoa.gr (S.P.); jvlamis@med.uoa.gr (J.V.); 2 2nd Orthopaedic Department, KAT General Hospital, 14561 Athens, Greece; marab.ortho@gmail.com; 3 Microbiology Department, KAT General Hospital, 14561 Athens, Greece; astylianakis@hotmail.com; 4 Department of Pathophysiology, Laiko General Hospital, National and Kapodistrian University of Athens, School of Medicine, 11527 Athens, Greece

**Keywords:** dithiothreitol, sonication, implant-associated infections, orthopaedic implants

## Abstract

Orthopaedic implant-associated infections (OIAIs) represent a notable complication of contemporary surgical procedures, exerting a considerable impact on patient outcomes and escalating healthcare expenditures. Prompt diagnosis holds paramount importance in managing OIAIs, with sonication widely acknowledged as the preferred method for detecting biofilm-associated infections. Recently, dithiothreitol (DTT) has emerged as a potential substitute for sonication, owing to its demonstrated ability to impede biofilm formation. This study aimed to compare the efficacy of DTT with sonication in identifying microorganisms within implants. Conducted as a prospective cohort investigation, the study encompassed two distinct groups: patients with suspected infections undergoing implant removal (Group A) and those slated for hardware explantation (Group B). Hardware segments were assessed for biofilm-related microorganisms using both sonication and DTT, with a comparative analysis of the two methods. A total of 115 patients were enrolled. In Group A, no statistically significant disparity was observed between DTT and sonication. DTT exhibited a sensitivity of 89.47% and specificity of 96.3%. Conversely, in Group B, both DTT and sonication fluid cultures yielded negative results in all patients. Consequently, this investigation suggests that DTT holds comparable efficacy to sonication in detecting OIAIs, offering a novel, cost-effective, and readily accessible diagnostic modality for identifying implant-associated infections.

## 1. Introduction

The orthopaedic surgical field has expanded rapidly with the advent of newer techniques and optimally designed implants aiming to restore patients’ physiological function by replacing a destroyed joint (joint arthroplasty) or reinforcing a damaged bone (internal or external fixation).

Minimally invasive procedures as well as robotic surgery have bloomed during the last decades, and implant related surgeries are now performed with increased precision and fewer complications. Bioengineering technology has evolved to provide dedicated implants for minimally invasive approaches with good long-term outcomes [1]. Additionally, customised implants, specifically designed for each patient, are a promising tool, although their implementation has not been established yet due to studies showing contradictory results [2].

Nonetheless, orthopaedic implants may fail to fulfill their expected functional outcomes due to complications. Loosening is a major one and can be either aseptic or septic. In the latter form, bacterial growth on the surface of the device is the most commonly implicated mechanism. Methods to overcome these kinds of complications have been developed with different implant surface materials and coatings offering theoretical advantages in mitigating bacterial colonisation. Metals, ceramics, and polymers are the materials most commonly used—alone or as alloys—in implant manufacturing, allowing for different mechanical and biochemical advantages over the other. Surface roughness, device design, implant coating, and addition of antibiotic-loaded cement between the implant and the bone are some of the factors associated with bacteria adhesion and colonisation. Even so, these favourable properties have been shown in in vitro or in vivo studies and not yet in well-powered clinical studies [3,4].

The number of total joint replacements and internal or external fixations performed annually has increased worldwide, and thus, implant related complications, including implant-associated infections, have become more common [5,6]. Patients are confronted with prolonged hospitalisations, longtime antimicrobial treatment, and revision surgery, all of which are associated with a higher risk of morbidity, mortality, and healthcare costs [7]. The risk of infection after joint replacement ranges between 0.3 and 1.9% [8], after internal fixation an infection occurs in approximately 5% [9], while after external fixation, an infection has been reported to lie between 3% and 80% [10].

Specific infection control measures have been recommended to address orthopaedic implant-associated infections including, among others, perioperative antibiotic administration, antiseptic washing quality of both the staff and the patient before surgery, and behavioural optimisation strategies in the operating room [11].

Implant-related infections may be caused by direct wound contamination or can be haematogenous in origin. The majority of those though—and most difficult to be addressed—are biofilm-related. A mature biofilm is a well-organised structure containing a mixture of microbes, polysaccharides, proteins, and DNA. Biofilms adhere to a surface and offer a shield of protection to the microorganisms they host, rendering them undetectable with conventional cultures [12].

Alternative techniques, such as sonication, have been developed to dislodge the adherent bacteria from the implant surface. Sonication fluid cultures have been found in multiple studies to improve microbiological diagnostic accuracy [13,14]. Although this procedure still represents the gold standard for the disruption of the prostheses biofilm and the enhancement of bacterial growth, the necessary equipment is not available in all laboratories. Recently, the use of dithiothreitol (DTT) has been shown to inhibit biofilm formation [15]. The aim of this study was to evaluate the effectiveness of using dithiothreitol in comparison to sonication fluid culture for identifying the culprit organisms that cause infection in patients with suspicion of such implant-related infections.

## 2. Materials and Methods

A prospective cohort study was conducted at our institution between 2018 and 2022. Delays in recruitment, acquisition, and analysis of samples compared to initial protocol schedule were attributed to the COVID-19 pandemic.

### 2.1. Patients

**Figure 1:**
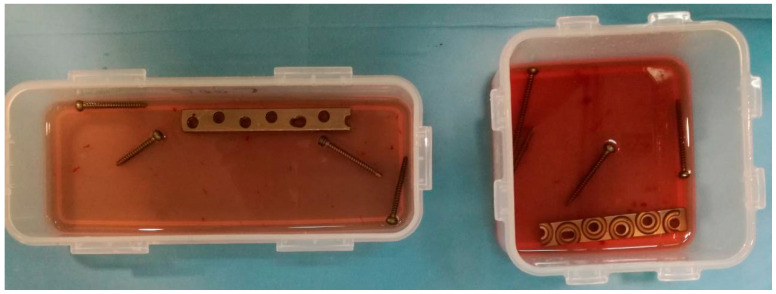
Hardware aseptically removed and divided in two equal-length segments and each segment placed into separate sterile solid air-tight containers.

Consecutive patients undergoing implant removal either due to suspicion of implant-related infection or scheduled removal of metalwork were included. Patients enrolled in this study were divided into two groups. Group A consisted of patients with suspicion of implant-related infection, while Group B (control group) consisted of patients with scheduled removal of metalwork, such as syndesmotic screws. Patients in Group B had no symptom or sign of infection. Suspicion of infection was based on the presence of one of the following: CRP > 10 mg/L and/or ESR > 30 mm/h, signs of inflammation (pain, reduced range of motion, erythema, fever), fistula, or pus. Intraoperatively, the hardware was aseptically removed and was divided in two equal-length segments for all patients. Each segment was placed into a separate sterile solid air-tight container, and then the two containers were transported to the laboratory where they were processed within 6 h (Figure 1). The implant segment from the first container was processed with sonication, and the implant segment from the second container with DTT.

### 2.2. Techniques

In the sonication method, a low-frequency and intensity ultrasound was performed in a special ultrasonic water bath (BactoSonic, Bandelin, Berlin, Germany), as described by Trampuz et al. [16]. Small-sized implants were covered entirely inside the special container with Ringer’s solution or 0.9% NaCl, while big-sized implants had only a part of them—ranging from 80–90%—covered with one of the aforementioned solutions. Subsequently, the containers with implants were vortexed for 30 s, followed by sonication at a frequency of 35–40 kHz for 1 min. After sonication, additional vortexing was performed for 30 s. This was followed by quantitative inoculation with 100 μLof sonication fluid in a blood agar plate for aerobic culture at 37 °C for 5 days, in a chocolate agar plate in 5% CO_2_ at 37 °C for 10 days, and in a brucella agar plate for anaerobic culture at 37 °C for 15 days. Furthermore, 3–4 mL of ultrasound fluid was added in thioglycolate broth at 37 °C. If no growth was observed in the plates, then the thioglycolic broth was grown in blood and brucella agar plates. The drills were inspected daily for microbial growth [7]. All samples were handled in a bio-safety chamber to avoid contamination.

In the second container, an alternative approach was performed to dislodge bacteria from infected implants. The implant was immersed in a phosphate-buffered solution of DTT concentration 6.48 mM (0.1% *w*/*v* DTT in phosphate-buffered saline) and stirred mechanically for 15 min at room temperature. Quantitative culture of the solution was then performed in similar conditions, as mentioned before for ultrasound fluid. Macroscopically, different microbial colonies were isolated, identified, and tested for antimicrobial susceptibility by using VITEK Compact (Biomerieux, Marcy l’Etoile, France). Sonication or DTT fluid cultures were considered positive if at least five colonies grew on agar plates after 24 h of incubation for aerobes and fungi, and up to 14 days of incubation for anaerobes. Samples with suspected contamination were excluded from the study.

### 2.3. Statistics

Demographic and epidemiological data were recorded in all patients. Informed consent was obtained from all participants included in the study, and all procedures were in accordance with the ethical standards of the institutional research committee (protocol code: 15239 and date of approval: 18 December 2017). The primary outcome of our study was to evaluate the accuracy of DTT versus sonication, the gold standard method, in the detection of implant-related infections by calculating DTT’s sensitivity and specificity with 95% confidence intervals (CIs) in Group A. Positive predictive value (PPV), negative predictive value (NPV), and overall accuracy of DTT fluid culture in Group A were the secondary outcomes. The two methods were compared using McNemar’s test. Differences were considered significant when *p* values were equal to or less than 0.05. A sample size of 65 patients was deemed adequate to attain a statistical power of 80% and an α value of 0.05 in the study. All calculations were performed using the statistical software package SPSS version 16.0 (SPSS Chicago, IL, USA).

## 3. Results

Samples from 116 patients were collected. One patient was excluded from the study because one of the containers was not sealed appropriately. Finally, a total of 115 patients were enrolled in the study, 65 in Group A and 50 in Group B. Patients’ demographics for each group are summarised in Table 1.

In group A, both techniques (sonication and DTT) were positive for 34 patients and negative for 26 patients. Among discordant results, in four cases, microbial growth was observed in sonication fluid cultures with DTT being negative, while only in one case sonication was negative with DTT being positive (Table 2).

McNeamar’s paired test revealed no significant difference between the two methods (*p*= 0.3711, 95% CI: 0.005 to 2.526). Assuming sonication as a gold standard method for biofilm disruption and identification of microorganisms of implant-related infections, the sensitivity of DTT fluid culture was 89.47% (95% CI 75.20–97.06%) and the specificity was 96.3% (95% CI: 81.03–99.91%).

PPV, NPV, and overall accuracy of DTT fluid culture in Group A were 97.14% (95% CI: 83.20–99.57%), 86.67% (95% CI: 71.95–94.28), and 92.31% (95% CI: 82.95–97.46), respectively.

Among patients with scheduled explantation of metalwork (Group B—control), no bacterial growth was observed in cultures, and all the results were negative with both techniques—sonication and DTT. Thus, concordance between the two methods was 100% in this group.

The total concordance between the two methods in both groups was 95.65% (110/115 cases, Table 3).

As presented in Table 4, Gram-positive cocci were the most frequently isolated bacteria. Specifically, *Staphylococcus epidermidis* was isolated from 15 sonicated samples and 13 DTT-treated samples. *Staphylococcus aureus* was found in 10 and 11 samples treated with sonication and DTT, respectively. *Staphylococcus haemolyticus* was found in both sonicated and DTT-treated cultures in seven cases. In addition, we had three cases where *Staphylococcus hominis* was identified in sonication fluid cultures but in only one of them DTT achieved detection of the microorganism. Finally, *Staphylococcus lugdunensis*, *Staphylococcus capitis,* and *Enterococcus faecalis* were isolated once each.

## 4. Discussion

Orthopaedic procedures using implants have increased over time with a rise in subsequent complications such as orthopaedic implant-associated infections [17,18]. Early diagnosis and identification of the causative agent are crucial for effective treatment and management. Considering that low-grade infection is the reason for aseptic loosening in many cases, clinical evaluation is insufficient for the accurate diagnosis of infection, and a reliable microbiological diagnostic method is necessary [19]. Periprosthetic tissue culture constitutes a conventional method of diagnosis that can detect and identify organisms adhered to the prosthesis and is comparable to contemporary techniques when an appropriate tissue culture protocol is used [20]. However, tissue cultures can be negative either in the case of previous antibiotic therapy or in the case of infections caused by less virulent organisms organised within biofilms [21,22]. Biofilm is a consortium of microorganisms enclosed in an extracellular matrix mainly consisting of polysaccharides, proteins, and eDNA [23,24]. Microbes cannot be detected in periprosthetic tissue specimens since the prosthesis surface, where the biofilm is formed, is not sampled [25]. The extracellular polymeric substances (EPS) matrix creates an antibacterial barrier to antibiotics and makes biofilms resistant to antimicrobial agents at concentrations of up to 1000 times of that needed to inactivate genetically equivalent planktonic bacteria [26]. Given that, the proper therapeutic protocol of an implant-associated infection includes the explantation of the infected hardware. In the case of a healed fracture, that could be achieved with surgical debridement, implant removal, and appropriate antibiotic treatment [27], while in the case of periprosthetic joint infection, a two-stage procedure is required [13]. The infected implant is replaced with an antibiotic-loaded cement spacer, antibiotic therapy is administered, and a second-stage revision surgery is performed when the infection has been cured. Conventional tissue cultures have inherent flaws to detect bacteria embedded in biofilms [28], thus a technique with the capacity to disrupt biofilm is necessary. So far, sonication constitutes the gold standard technique for biofilm disruption and is a validated method for detection of implant-associated infections [14]. However, this method is not used for tissue specimens and has some limitations such as the necessity of dedicated laboratory tools and a considerable risk of contamination, due to possible damages or inaccurate sealing of samples’ containers, size of explanted prostheses, and bacteria proliferation in sonication water [29]. Thus, the identification and validation of an alternative reliable and readily available method would be of prime importance. Wu et al. have reported that DTT, a sulfhydryl compound commonly used in pathology laboratories, is able to inhibit biofilm formation in vitro [15]. As with sonication, DTT has been shown to be superior to tissue cultures for the identification of implant-associated infections [30]. Furthermore, a recent study from Italy [31] compared the use of DTT versus sonication in patients with aseptic loosening and periprosthetic joint infection. DTT showed the same specificity (94.1%) to sonication but higher sensitivity (85.7% vs. 71.4%). However, in this study, these two techniques were performed at different implant parts of the same patient, assuming that the infection would affect both components of a total joint arthroplasty. Processing different parts of an arthroplasty with different techniques—for example, in a hip arthroplasty processing the acetabular component with sonication and the stem with DTT—assumes that any infection is localised in both components. The qualitative characteristics of the bacteria as well as the bacterial load may nonetheless differ between these two parts, and thus important information might be missed. In another in vitro pilot study published from the same institute [32], Drago et al. processed polyethylene and titanium discs, covered by biofilm, with DTT and sonication and found that mean colony counts were similar with both techniques. In contrast to the above outcomes, a recent study [33] compared the activity of chemical methods (EDTA and DTT) for biofilm dislodgement to sonication in an in vitro model of artificial biofilm and demonstrated that sonication is superior to the chemical methods for dislodgement of bacterial biofilms of *S. epidermidis*, *S. aureus*, *E. coli,* and *P. aeruginosa* from the artificial surface. Higher CFU counts were detected with the sonication technique rather than the EDTA or DTT method with all tested microorganisms. Sambri et al. [34] have also recently assessed the effectiveness of DTT in comparison to sonication for the diagnosis of prosthetic joint infections. In this randomised study, 232 patients undergoing revision knee or hip arthroplasty were allocated to sonication or DTT. The authors concluded that there was no difference in sensitivity between DTT and sonication for the detection of prosthetic joint infections, and both of those tests were more sensitive than standard tissue cultures. Finally, a recent meta-analysis pooling together data from more than 700 implants compared the diagnostic accuracy of sonication versus DTT in patients undergoing total hip or knee arthroplasty. The authors did not find any differences between the two methods after adjusting for baseline antibiotic use and study quality, in accordance with our results [35].

In our study, we assessed the efficacy of DTT compared with a gold standard technique to detect implant-associated infections and also tried to identify the causative agent, as it is accepted that through both DTT and sonication, bacteria can be dislodged from biofilms and then can be easily cultured. The explanted materials were processed with both sonication and DTT techniques, and the results were comparable. This is the first, to our knowledge, study with a direct comparison between the two methods at the same implant. The explanted hardware was divided into two equal-length parts, and each sample was allocated either to the sonication or DTT method. Both sonication and DTT did not detect implant colonisation among healthy control group patients whose hardware was removed for routine reasons. We believe that the reason for these results is the strictly aseptic and standardised technique used in the sterile environment of a surgical room during the removal of these implants, decreasing the risk of contamination. A low bacterial load unable to be detected by either sonication or DTT could be an alternative reason for negative results, although the rate of such false negative results with these methods is quite low [36]. This would be more commonly expected in healthy controls without symptoms of an infection and with an intact immune system able to mount a strong immune response to prevent a clinically significant infection.

In an era where multidrug-resistant organisms have emerged as a major health system threat and cost effectiveness is a driver in health infrastructure-related decisions, a reliable and cheap method to identify causative microbes of orthopaedic implant-related infections is, in our opinion, of paramount importance.

## 5. Conclusions

The results of our study showed that the DTT technique is non-inferior in comparison to the sonication technique and is an alternative option obviating the need for specialised equipment needed for sonication. Further studies with a larger sample size are required to validate our findings. In conclusion, DTT represents a new, non-expensive, and readily available diagnostic method for detection of implant-associated infections.

## Figures and Tables

**Table 1:** Patients’ demographic data.

	Group A	Group B	Total
N (%)	65 (56.5)	50 (43.5)	115 (100)
Age (yr, mean ± SD)	57.13 ± 18.19	55.72 ± 19.01	
Gender (male/female)	40/25	27/23	67/48
Patients with diabetes, N (%)	7 (10.76)	8 (16)	15 (13.04)
Patients with rheumatoid arthritis, N (%)	6 (9.23)	1 (2)	7 (6.09)

**Table 2:** Comparison of sonication vs. DTT fluid culture in patients with suspicion of infection (Group A).

	DTT (+)	DTT (−)	Total
Sonication (+)	34	4	38
Sonication (−)	1	26	27
Total	35	30	65

**Table 3:** Comparison between sonication and DTT technique.

Method	Group	Positive	Negative
Sonication	A	38	27
	B	0	50
DTT	A	35	30
	B	0	50

**Table 4:** Microorganisms isolated with each technique.

Microorganism	Sonication	DTT
*Staphylococcus epidermidis*	15	13
*Staphylococcus aureus*	10	11
*Staphylococcus haemolyticus*	7	7
*Staphylococcus hominis*	3	1
*Staphylococcus lugdunensis*	1	1
*Staphylococcus capitis*	1	1
*Enterococcus faecalis*	1	1

## Data Availability

Data relevant to this study are included in this manuscript. Additional data can be requested from the corresponding author.

## References

[1] Toyoda S., Kaneko T., Mochizuki Y., Hada M., Takada K., Ikegami H., Musha Y. (2021). Minimally invasive surgery total knee arthroplasty is less popular, but the prosthesis designed specifically for MIS provides good survival and PROMs with a minimum follow-up of 10 years. J. Orthop. Surg. Res..

[2] Benignus C., Buschner P., Meier M.K., Wilken F., Rieger J., Beckmann J. (2023). Patient Specific Instruments and Patient Individual Implants-A Narrative Review. J. Pers. Med..

[3] Bohara S., Suthakorn J. (2022). Surface coating of orthopedic implant to enhance the osseointegration and reduction of bacterial colonization: A review. Biomater. Res..

[4] Szczęsny G., Kopec M., Politis D.J., Kowalewski Z.L., Łazarski A., Szolc T. (2022). A Review on Biomaterials for Orthopaedic Surgery and Traumatology: From Past to Present. Materials.

[5] Kennedy D.G., O’Mahony A.M., Culligan E.P., O’Driscoll C.M., Ryan K.B. (2022). Strategies to Mitigate and Treat Orthopaedic Device-Associated Infections. Antibiotics.

[6] Davidson D.J., Spratt D., Liddle A.D. (2019). Implant materials and prosthetic joint infection: The battle with the biofilm. EFORT Open Rev..

[7] Tani S., Lepetsos P., Stylianakis A., Vlamis J., Birbas K., Kaklamanos I. (2018). Superiority of the sonication method against conventional periprosthetic tissue cultures for diagnosis of prosthetic joint infections. Eur. J. Orthop. Surg. Traumatol..

[8] Evangelopoulos D.S., Stathopoulos I.P., Morassi G.P., Koufos S., Albarni A., Karampinas P.K., Stylianakis A., Kohl S., Pneumaticos S., Vlamis J. (2013). Soni-cation: A valuable technique for diagnosis and treatment of periprosthetic joint infections. Sci. World J..

[9] Darouiche R.O. (2004). Treatment of infections associated with surgical implants. N. Engl. J. Med..

[10] Jennison T., McNally M., Pandit H. (2014). Prevention of infection in external fixator pin sites. Acta Biomater..

[11] Marche B., Neuwirth M., Kugler C., Bouillon B., Mattner F., Otchwemah R. (2021). Implementation methods of infection prevention measures in orthopedics and traumatology—A systematic review. Eur. J. Trauma Emerg. Surg..

[12] Nguyen L.L., Nelson C.L., Saccente M., Smeltzer M.S., Wassell D.L., McLaren S.G. (2002). Detecting bacterial colonization of implanted orthopaedic devices by ultrasonication. Clin. Orthop. Relat. Res..

[13] Liu H., Zhang Y., Li L., Zou H. (2017). The application of sonication in diagnosis of periprosthetic joint infection. Eur. J. Clin. Microbiol. Infect. Dis..

[14] Rothenberg A.C., Wilson A.E., Hayes J.P., O’Malley M.J., Klatt B.A. (2017). Sonication of Arthroplasty Implants Improves Accuracy of Periprosthetic Joint Infection Cultures. Clin. Orthop. Relat. Res..

[15] Wu X., Wang Y., Tao L. (2011). Sulfhydryl compounds reduce staphylococcus aureus biofilm formation by inhibiting pia biosynthesis. FEMS Micro-Biol. Lett..

[16] Trampuz A., Piper K.E., Jacobson M.J., Hanssen A.D., Unni K.K., Osmon D.R., Mandrekar J.N., Cockerill F.R., Steckelberg J.M., Greenleaf J.F. (2007). Sonication of removed hip and knee prostheses for diagnosis of infection. N. Engl. J. Med..

[17] Ariza J., Cobo J., Baraia-Etxaburu J., Benito N., Bori G., Cabo J., Corona P., Esteban J., Horcajada J.P., Lora-Tamayo J. (2017). Executive summary of management of prosthetic joint infections. Clinical practice guidelines by the Spanish Society of Infectious Diseases and Clinical Microbiology (SEIMC). Enferm. Infecc. Microbiol. Clin..

[18] Basile G., Gallina M., Passeri A., Gaudio R.M., Castelnuovo N., Ferrante P., Calori G.M. (2021). Prosthetic joint infections and legal disputes: A threat to the future of prosthetic orthopedics. J. Orthop. Traumatol..

[19] Heinz N.R., Clement N.D., Young R.N., Duckworth A.D., White T.O., Molyneux S.G. (2023). Rate and factors associated with surgical site infection following aseptic revision fixation of orthopaedic trauma injuries. Eur. J. Orthop. Surg. Traumatol..

[20] Vasoo S. (2018). Improving the Diagnosis of Orthopedic Implant-Associated Infections: Optimizing the Use of Tools Already in the Box. J. Clin. Microbiol..

[21] Berbari E.F., Marculescu C., Sia I., Lahr B.D., Hanssen A.D., Steckelberg J.M., Gullerud R., Osmon D.R. (2007). Culture-negative prosthetic joint infec-tion. Clin. Infect. Dis..

[22] Yoon H.K., Cho S.H., Lee D.Y., Kang B.H., Lee S.H., Moon D.G., Kim D.H., Nam D.C., Hwang S.C. (2017). A Review of the Literature on Culture-Negative Periprosthetic Joint Infection: Epidemiology, Diagnosis and Treatment. Knee Surg. Relat. Res..

[23] Xu Q., Hu X., Wang Y. (2021). Alternatives to Conventional Antibiotic Therapy: Potential Therapeutic Strategies of Combating Antimicrobial-Resistance and Biofilm-Related Infections. Mol. Biotechnol..

[24] Zhao A., Sun J., Liu Y. (2023). Understanding bacterial biofilms: From definition to treatment strategies. Front. Cell Infect. Microbiol..

[25] Puchner S.E., Döring K., Staats K., Böhler C., Lass R., Hirschl A.M., Presterl E., Windhager R., Holinka J. (2017). Sonication culture improves microbiological diagnosis of modular megaprostheses. J. Orthop. Res..

[26] Abebe G.M. (2020). The Role of Bacterial Biofilm in Antibiotic Resistance and Food Contamination. Int. J. Microbiol..

[27] Maniar H.H., Wingert N., McPhillips K., Foltzer M., Graham J., Bowen T.R., Horwitz D.S. (2016). Role of sonication for detection of infection in explanted orthopaedic trauma implants. J. Orthop. Trauma.

[28] Gbejuade H.O., Lovering A.M., Webb J.C. (2015). The role of microbial biofilms in prosthetic joint infections. Acta Orthop..

[29] Perry K.I., Hanssen A.D. (2017). Orthopaedic Infection: Prevention and Diagnosis. J. Am. Acad. Orthop. Surg..

[30] Giannetti A., Romano J., Fidanza A., Di Mauro M., Brunetti M., Fascione F., Calvisi V. (2022). The diagnostic potential of MicroDTTect compared to conventional culture of tissue samples in orthopedic infections. Lo Scalpello J..

[31] Drago L., Signori V., De Vecchi E., Vassena C., Palazzi E., Cappelletti L., Romano D., Romano C.L. (2013). Use of dithiothreitol to improve the diagnosis of prosthetic joint infections. J. Orthop. Res..

[32] Drago L., Romanò C.L., Mattina R., Signori V., De Vecchi E. (2012). Does dithiothreitol improve bacterial detection from infected prostheses? A pilot study. Clin. Orthop. Relat. Res..

[33] Karbysheva S., Di Luca M., Butini M.E., Winkler T., Schütz M., Trampuz A. (2020). Comparison of sonication with chemical biofilm dislodgement methods using chelating and reducing agents: Implications for the microbiological diagnosis of implant associated infection. PLoS ONE.

[34] Sambri A., Cadossi M., Giannini S., Pignatti G., Marcacci M., Neri M.P., Maso A., Storni E., Gamberini S., Naldi S. (2018). Is Treatment with Dithiothreitol More Effective Than Sonication for the Diagnosis of Prosthetic Joint Infection?. Clin. Orthop. Relat. Res..

[35] Tsikopoulos K., Christofilos S.I., Kitridis D., Sidiropoulos K., Stoikos P.N., Gravalidis C., Givissis P., Papaioannidou P. (2022). Is sonication superior to dithiothreitol in diagnosis of periprosthetic joint infections? A meta-analysis. Int. Orthop..

[36] Drago L., Romanò D., Fidanza A., Giannetti A., Erasmo R., Mavrogenis A.F., Romanò C.L. (2023). Dithiotreitol pre-treatment of synovial fluid samples improves microbiological counts in peri-prosthetic joint infection. Int. Orthop..

